# The Role of Temperature and Subphase Components in Shaping Selected Physicochemical Properties of the Phosphatidylinositol Monolayer

**DOI:** 10.3390/ijms26083472

**Published:** 2025-04-08

**Authors:** Iwona Golonka, Izabela W. Łukasiewicz, Aleksandra Sebastiańczyk, Katarzyna E. Greber, Wiesław Sawicki, Witold Musiał

**Affiliations:** 1 Department of Physical Chemistry and Biophysics, Wroclaw Medical University, Borowska 211A, 50-556 Wroclaw, Poland; iwona.golonka@umw.edu.pl (I.G.); izabela.w.lukasiewicz@student.umw.edu.pl (I.W.Ł.); aleksandra.sebastianczyk@student.umw.wroc.pl (A.S.); 2 Department of Physical Chemistry, Faculty of Pharmacy, Medical University of Gdańsk, Al. Gen. J. Hallera 107, 80-416 Gdańsk, Poland; katarzyna.greber@gumed.edu.pl (K.E.G.); wieslaw.sawicki@gumed.edu.pl (W.S.)

**Keywords:** compressibility coefficient, compression reversibility factor, antibacterial peptides, ascorbic acid, 3-O-ethyl-L-ascorbic acid, phosphatidylinositol membrane

## Abstract

*Acne vulgaris* is one of the most common skin diseases, and its development is closely linked to the overgrowth of the bacterium *Cutibacterium acnes*. More than half of the strains of this bacterium are resistant to antibiotics, which has prompted scientists to look for alternatives, such as antibacterial peptides, that can replace traditional drugs. Due to its antioxidant properties, ascorbic acid may be a promising ally in the treatment of acne. The aim of our study was to evaluate the effect of peptide (KWK)_2_-KWWW-NH_2_ **(P5)** in the presence of ascorbic acid (AA) and its derivative (3-O-ethyl-L-ascorbic acid, EAA) on the stability and organization of phosphatidylinositol monolayers (PI) at temperatures of 25–35 °C. This study showed that the monolayers were in the expanded liquid state (35.28–49.95 mN/m) or in the transition between the expanded liquid and condensed phases (51.50–57.49 mN/m). Compression and decompression isotherms indicated the highest flexibility of the PI + P5 system, where the compression reversibility coefficient of isotherm values ranged from 80.59% to 97.77% and increased for each loop with increasing temperature. At 35 °C, the surface pressure of the monolayer in the PI + P5, PI + P5 + AA and PI + P5 + EAA systems changed less with time.

## 1. Introduction

*Acne vulgaris*, as a multifactorial disease, is influenced by many factors, such as genetic predisposition [1], hormonal activity [2], overproduction of sebum in the sebaceous glands [3], comedogenicity [4], *Propionibacterium acnes* [5] and diet [6]. Stress increases cortisol and androgen levels, which can increase sebum secretion and the symptoms of acne [7]. In addition, air pollutants such as dust, smog and other chemicals can contribute to the development of acne [8]. The misuse and overuse of antibiotics have significantly reduced their effectiveness, leading to an increase in the number of resistant microorganisms. One promising solution in the fight against drug resistance is the use of antimicrobial peptides (AMPs), which are characterized by a broad spectrum of action [9,10]. AMPs are effective in combating *Gram-negative bacteria*, which are a more difficult target than *Gram-positive* bacteria. The outer cell membrane of *Gram-negative bacteria* acts as an additional barrier, impeding the penetration of many traditional antibiotics [11,12]. Most AMPs are characterized by a positive charge (from +2 to +13), hydrophobicity and amphipathicity [13]. Increasing the charge can improve their antibacterial activity. One example are magainin II derivatives: increasing the charge from +3 to +5 improved their efficacy, but further increasing the charge, e.g., to +7, led to increased hemolytic activity and a loss of antimicrobial properties [14]. Strong interactions of the peptide with the phospholipid head group may be responsible for the lack of translocation of the peptide to the inner layer of the membrane [15]. Peptides with an excessively low hydrophobicity may increase the probability of dimerization, which makes their access to the bacterial membrane difficult. Increasing this parameter, on the other hand, may allow for a deeper penetration of peptides into the hydrophobic core of the membrane [16]. The amphipathicity of peptides is based on the balance between cationic and hydrophobic residues, and its increase does not necessarily affect antimicrobial activity but may lead to increased hemolysis [17]. According to studies in the literature, different parameters play a unique role depending on the peptide sequence. The search for new therapies against skin infections is an ongoing and dynamic process. About 30% of people aged 11 to 30 years struggle with acne vulgaris, which is not merely an aesthetic problem. Acne can have serious psychological consequences, lowering the self-esteem and self-confidence of patients [18]. Modern peptides intended for topical use should have antimicrobial activity, minimal cytotoxicity and no, or highly limited, cytotoxic effects [19]. Additionally, they may benefit from antioxidant properties and resistivity to solar radiation [20].

**Figure 1:**
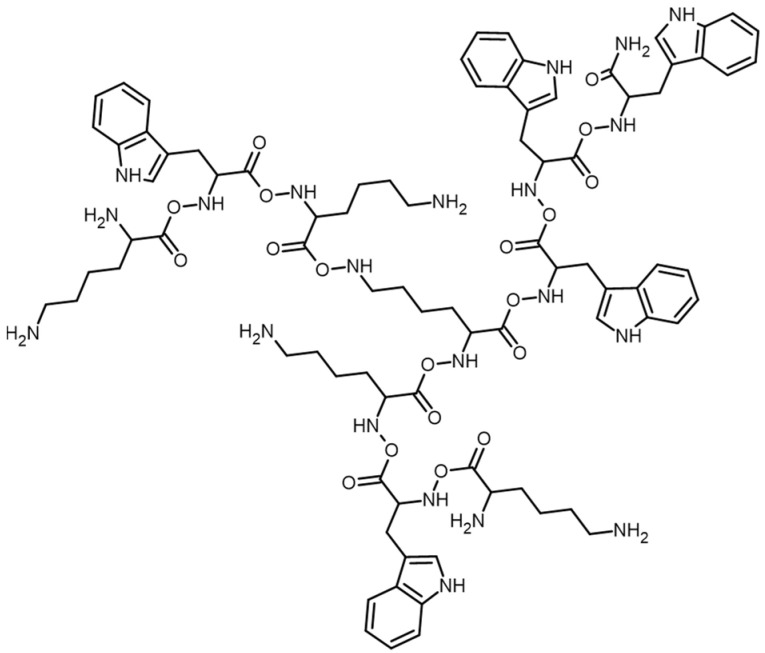
Structure of tested peptide P5 [21].

Peptide (KWK)_2_-KWWW-NH_2_, with a 4+ charge and a molar mass of 1588 g/mol, used in previous publications as P5 (Figure 1), showed antimicrobial activity against *Staphylococcus aureus*. The balanced number of polar lysine residues and nonpolar tryptophan residues in this peptide could contribute to its significantly higher antioxidant activity compared to its derivatives [21].

The antimicrobial activity of the peptide absorbed onto polymeric–bacterial cellulose (BC) produced by *Komagataeibacter xylinum* confirmed its potential use as a BC carrier against *S. aureus* and *C. acnes*. One of the hypotheses was that this peptide could disrupt the integrity of the bacterial membrane [21]. Substances with medium lipophilicity, of logP value in the 1.0–3.0 range, exhibit optimal penetration ability, and the compounds selected for our study met these criteria. Moreover, tryptophan residues in P5 show a high affinity for the interphase regions of biological membranes, suggesting that tryptophan can act as an anchoring molecule, binding the peptide to microbial cells. The concentration of the peptide used in the study, at 1000 mg/L, corresponded to the concentrations of octenidine- or polyhexanide-based antiseptics, commonly used in skin and wound care [22,23]. Studies have shown the lack of cytotoxicity of the P5 peptide towards fibroblast lines [21], which may support the wound healing process in vivo by supporting the function of fibroblasts [24]. The value of the diffusion coefficient of this compound was 0.0765 × 10^−5^ cm^2^/s. Due to the presence of nonpolar side chains, it forms a monolayer at the vacuum/water interface, which may be beneficial in the treatment of inflammation, creating a natural protective barrier between the damaged area and the external environment [25]. Data in the literature indicate that solar energy can modify the conformation of collagen molecules [26], but our results did not confirm the degradation of P5 bonds under the influence of solar radiation [21]. Phosphatidylinositol, a component of lipid membranes, plays a key role in cell function. Different substitutions of the inositol ring may affect cellular processes such as endocytosis, exocytosis, signaling, or the functioning of ion channels. Phosphatidylinositol, with triacylglycerol and other lipids, is part of the cell envelope of the bacteria *Cutibacterium acnes* [27]. Ascorbic acid (AA) may support acne therapy through anti-inflammatory effects, skin regeneration and reducing discoloration and acne scars [28,29]. AA increases the photostability of collagen in the UV range. UV radiation, via free radicals appearing in collagen, results in photodegradation; however, the macromolecules may be restored due to electron donation from ascorbic acid [30,31]. The rapid degradation of AA poses challenges for the local delivery of the molecule in cosmetic and pharmaceutical products. The physicochemical properties of AA, such as its melting point (190–192 °C), partition coefficient (log P(o/w) = −1.85) and dissociation constant (pKa = 4.25), are not optimal for transport through the skin [32]. Therefore, in cosmetic products, 3-O-ethyl-l-ascorbic acid (EAA), a derivative of l-ascorbic acid with an ethyl group on the third carbon atom, is used. This structural modification protects the 3-OH group from ionization and thus the molecule from oxidation, but it also causes changes in EAA’s physicochemical properties. The melting point, log P(o/w) value and pKa value of EAA were determined to be 114.39 ± 0.5 °C, −1.07 ± 0.03 and 7.72 ± 0.01, respectively. The penetration of EAA through the skin was observed with the following carriers: 1.2-hexanediol, glycerol, propylene glycol, 1.2-pentanediol, isopropyl alcohol, propylene glycol monolaurate and propylene glycol monocaprylate [33]. The temperature of human skin reflects heat exchange conditions at the body–environment boundary [34]. The skin’s temperature acquires different values depending on the region of the face, the with highest values found on the forehead and the lowest on the surface of the ears [35]. A bilateral symmetry of cheek and ear temperature has been observed [36].

The aim of this study was to assess the influence of P5 peptide, ascorbic acid and 3-O-ethylascorbic acid in the aqueous subphase on the physicochemical properties of the phosphatidylinositol monolayer at temperatures of 20 °C to 35 °C using the Langmuir method. The obtained results may provide an answer to the question of which of the oligopeptides studied by the authors build into or destabilize the model membrane and thus could potentially support the treatment of acne. In a previous work, the authors noticed that, for example, adding AA to the subphase caused a faster phase transition in most of the tested systems. On the other hand, adding EAA to the subphase where the peptides ((WKWK)_2_-KWKWK-NH_2_ or (C12)_2_-KKKK-NH_2_) were already present resulted in a decrease in surface pressure of the phosphatidylinositol monolayer over time [37].

This research study aimed to select important factors affecting the stability, organization and molecular interactions of model biological membranes. Reports in the literature confirm temperature’s effect on the fluidity of membranes and thus on the environmental interactions of other components of the structures. The data may support the design of effective anti-acne preparations based on innovative molecules.

## 2. Results and Discussion

### 2.1. Compression Isotherms of PI Monolayer Assessed over Aqueous Subphase with Peptide and AA or EAA

The phosphatidylinositol (PI) monolayer was applied on two types of subphase containing varied P5 and AA or EAA (Table 1). Subsequently, the compression isotherms were recorded.

**Figure 2:**
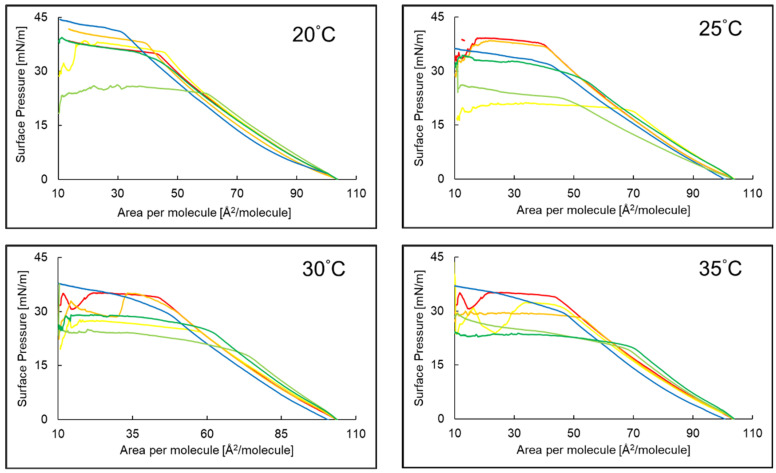
The course of the compression isotherms of the PI (**—**)*, PI + AA (**—**)*, PI + EAA (**—**)*, PI + P5 (**—**), PI + P5 + AA (**—**), PI + P5 + EAA (**—**) systems at a temperature of 20–35 °C. * Reference [37] reports a former evaluation of monolayers 1–3, which were reassessed for comparison purposes.

An increase in surface pressure above zero was observed at all four temperatures for a surface area per particle of about 103.65 Å^2^/molecule for the PI monolayer over the subphases with P5 and EAA or AA (Figure 2). The presence of P5 in the subphase resulted in a shift in the respective surface pressure values towards lower values of area per molecule. It was revealed that the collapse of the PI monolayers over the subphases with P5 and EAA occurs at a higher surface pressure than the collapse of the PI monolayer over the P5 and AA subphase at 20 °C, 25 °C and 30 °C. At 35 °C, the reverse result was observed. For the PI + P5 + AA and PI + P5 + EAA systems at 20 °C, 30 °C and 35 °C, the respective surface pressure values shifted towards a larger area per molecule, and a plateau occurred at a lower surface pressure compared to the compression isotherm of the PI + P5 system.

Based on the π-A isotherm diagrams, the following parameters characterizing the Langmuir monolayers formed by the given layer-forming compounds were determined: A_lift-off_—the value of the surface area per molecule at which an increase in surface pressure (π) above π = 0 mN/ m is recorded; π_collapse_—the value of the surface pressure at which the monolayer collapses; A_collapse_—the surface area per molecule at which the monolayer collapses; χ (A_collapse_/A_lift-off_); a_LE/LC_—slope of the linear part of the π–A isotherm in the range of the dynamic increase in surface pressure observed on the π–A isotherm. The obtained results are presented in Table 2. The addition of P5 to the subphase resulted in a reduction in the values of the a_LE/LC_, A_lift-off_ and A_collaps_ parameters of the PI monolayer in most cases compared to the results for the PI monolayer in the aqueous subphase. The opposite phenomenon was observed for the results of the π_collaps_ parameter. For the PI + P5 system at the four temperatures, the addition of both AA and EAA resulted in an increase in the values of the A_lift-off_, A_collapse_ and χ parameters and a decrease in the π_collapse_ of the PI monolayer.

### 2.2. Compressibility Coefficient of PI Monolayer Assessed over Aqueous Subphase with Peptide, AA or EAA

**Figure 3:**
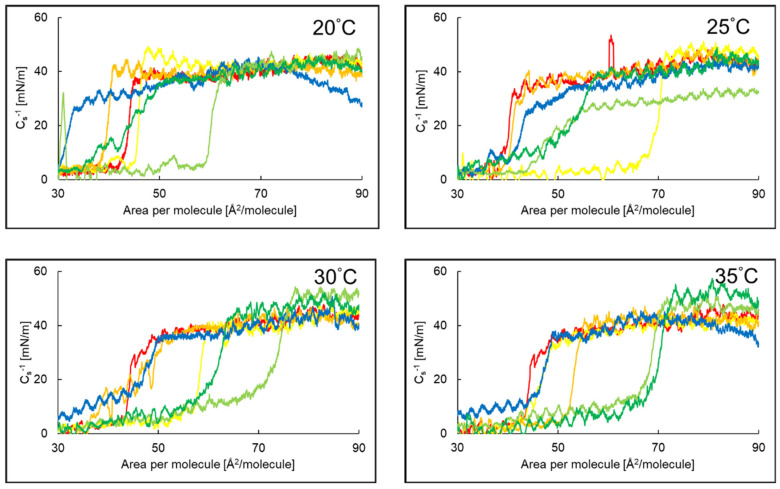
The compressibility coefficient as a function of the surface area per molecule of the PI (**—**)*, PI + AA (**—**)*, PI + EAA (**—**)*, PI + P5 (**—**), PI + P5 + AA (**—**), PI + P5 + EAA (**—**) systems at a temperature of 20–35 °C. * Reference [37] reports a former evaluation of monolayers 1–3, which were reassessed for comparison purposes.

Plots of the compressibility coefficient of the PI monolayer with AA, EAA or P5 in the water subphase as a function of the surface area per molecule at temperatures of 20 °C, 25 °C, 30 °C and 35 °C are shown in Figure 3. For temperatures of 20 °C and 30 °C, the curves of the PI + AA systems are shifted towards larger areas per molecule than for the PI + EAA systems or the PI monolayer alone on the water subphase. Adding the P5 peptide to the subphase containing AA or EAA molecules resulted in an even greater shift in the curves towards higher values of surface area per molecule for temperatures of 20 °C, 30 °C and 35 °C compared to the systems without the peptide.

Table 3 presents the compressibility coefficients for the different systems. The addition of AA or EAA to the subphase at 30 °C and 35 °C resulted in a decrease in the compressibility coefficient of the PI monolayer, and the opposite situation occurred at 20 °C and 25 °C. Comparing the C_S_^−1^ results for the PI monolayer and the PI system with P5 in the aqueous subphase, we observed a decrease in the C_S_^−1^ value after the addition of the peptide at all temperatures.

The addition of AA or EAA to the subphase of the PI + P5 system resulted in an increase in the compressibility coefficient at all temperatures, except for the PI + P5 + AA system at 25 °C. The value of the compressibility coefficient indicates that most of the layers in the systems were in the expanded liquid state or on the border of the expanded and condensed liquid state [38].

### 2.3. Compression and Expansion of PI Monolayer Assessed over Aqueous Subphase with Peptide, AA or EAA

**Figure 4:**
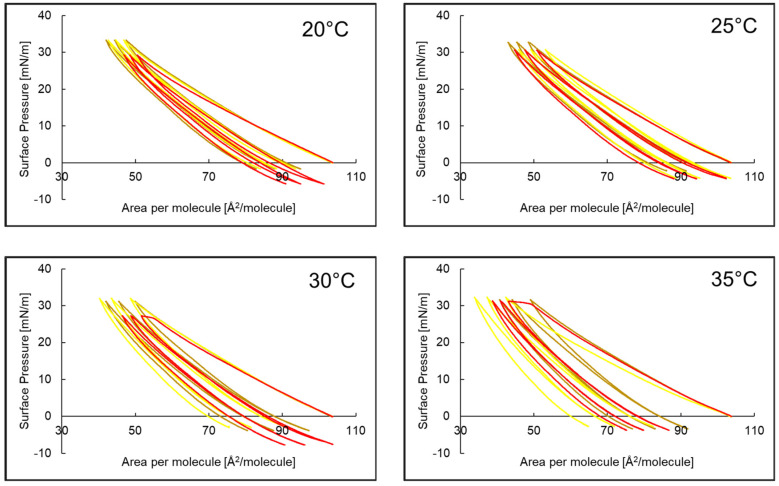
Hysteresis course of phosphatidylinositol PI monolayer (—)* with ascorbic acid PI + AA (—)* and 3-O-ethyl-ascorbic acid PI + EAA (—)* in the aqueous subphase at temperatures of 20–35 °C. * Reference [37] reports a former evaluation of monolayers 1–3, which were reassessed for comparison purposes.

The compression and decompression isotherms for the PI monolayer with AA or EAA in the aqueous subphase at temperatures of 20 °C, 25 °C, 30 °C and 35 °C are presented in Figure 4 as three hysteresis loops. At 20 °C and 25 °C, the hysteresis of the PI monolayers with EAA and AA in the aqueous subphase had a similar shape, and the width of each successive loop decreased slightly. At 30 °C and 35 °C, the hysteresis of PI monolayers with AA was clearly shifted towards smaller areas per molecule. At all temperatures, hysteresis was characterized by a wider first loop compared to the two subsequent loops. Decompression did not follow the compression path, and the maximum value of the surface pressure was 33.4 mN/m.

**Figure 5:**
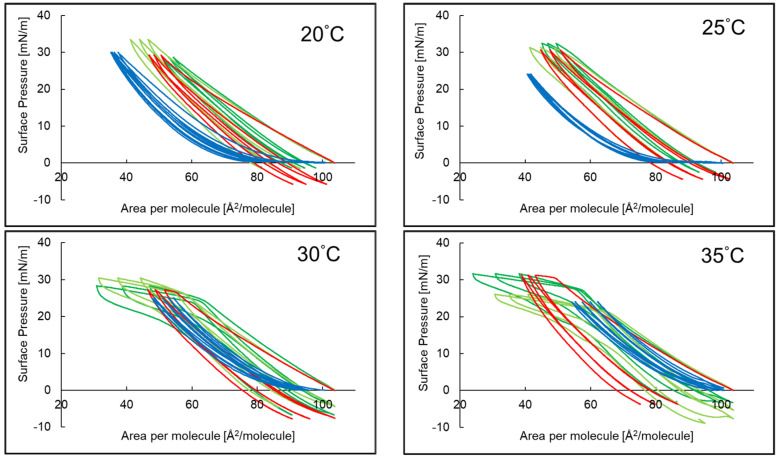
The hysteresis course of the phosphatidylinositol PI monolayer (—)* in the presence of P5; PI + P5 (—), ascorbic acid PI + P5 + AA (—) and 3-O-ethyl-ascorbic acid PI + P5 + EAA (—) in the aqueous subphase at temperatures of 20–35 °C. * Reference [37] reports a former evaluation of monolayers 1–3, which were reassessed for comparison purposes.

The course of the hysteresis of the PI monolayer with P5 and AA and EAA in the aqueous subphase is presented in Figure 5. At temperatures of 20 °C and 25 °C, the hysteresis loop of the PI + P5 system was shifted towards smaller areas per molecule compared to the system without the peptide, and the compression and decompression curves were close to each other. In the case of the PI monolayer and the PI + P5 + AA and PI + P5 + EAA systems, for both temperatures, the hysteresis was similar, with a slight shift in the three PI hysteresis loops towards smaller areas per molecule. At temperatures of 30 °C and 35 °C, in each system, the distance between compression and decompression increased for each loop. At a surface pressure of 20–30 mN/m, for these temperatures, a flattening of the loops occurs. The exception was the PI + P5 system, with a visibly narrower hysteresis loop, as compared to the former data.

Based on the values obtained from hysteresis measurements, the compression reversibility coefficient of the R_v_ isotherms was calculated, and the obtained results are presented in Table 4. The addition of AA to the subphase caused a change in the R_v_ parameter of loops 1, 2 and 3 of the hysteresis of the PI monolayer: an increase in the value for 20 °C, slight changes for 25 °C and a decrease in the value for 30 °C and 35 °C. When only P5 was present in the subphase, an increase in the R_v_ of the PI monolayer hysteresis loops 1, 2 and 3 was observed for each temperature, with the exception being loop 1 at 30 °C. For the PI + P5 system, adding both AA and EAA to the subphase caused a decrease in R_v_ for each PI monolayer hysteresis loop at 20–35 °C.

### 2.4. Surface Pressure of Evaluated Monolayers Versus Time in Assessed Systems

**Figure 6:**
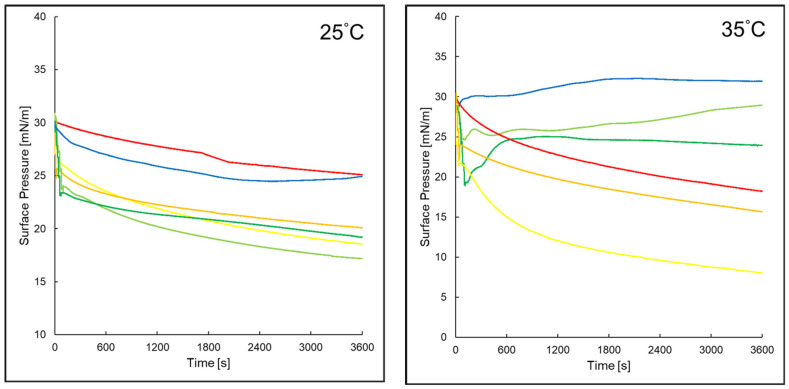
Surface pressure values of assessed layers versus time at temperatures of 25 °C and 35 °C of the tested systems: PI (**—**)*, PI + AA (**—**)*, PI + EAA (**—**)*, PI + P5 (**—**), PI + P5 + AA (**—**), PI + P5 + EAA (**—**) * Reference [37] reports a former evaluation of monolayers 1–3, which were reassessed for comparison purposes.

The influence of time laps on the surface pressure of the tested systems at 25 °C and 35 °C is shown in Figure 6. At 25 °C, a similarly decreasing course of surface pressure against time could be observed for all assessed systems except PI + P5. Monolayers of PI with EAA or AA in the subphase were characterized by a sudden decrease in surface pressure occurring when EAA or AA was added to the subphase. However, the curves became more flattened when time passed further. At 35 °C, monolayers of PI with P5 in the aqueous subphase showed an increase in and then a stabilization of surface pressure with time. After adding AA to the subphase, only at the beginning was a sudden decrease in surface pressure observed, followed by an increase and stabilization.

**Figure 7:**
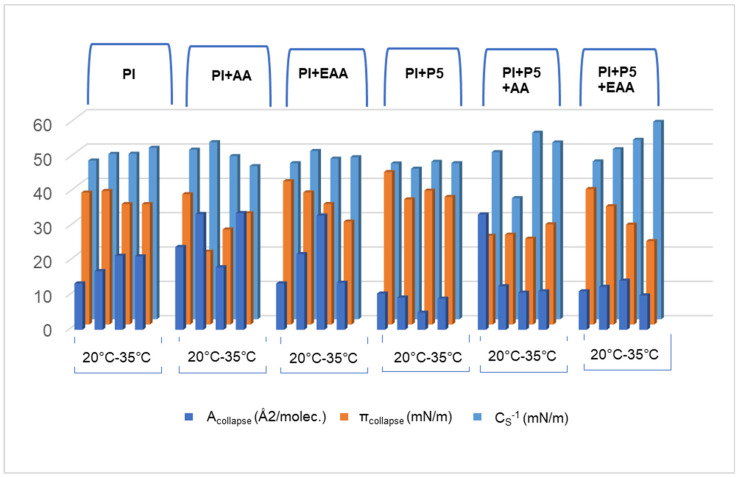
Parameter values of the tested systems at a temperature of 20–35 °C: A_collapse_ (Å^2^/molec.), π_collapse_ (mN/m), C_S_^−1^.

Due to the increasing resistance of *C. acnes* to the antibiotics used so far, new methods of treating its excessive growth are being developed [39]. According to our previous studies, peptides interact with the monolayers of phospholipid mixtures [25]. Thus, peptide (KWK)_2_-KWWW-NH_2_ was selected for the research presented in this work. Its effect on a monolayer composed of phosphatidylinositol [40] was assessed to check whether PI, as a meager fraction of bacterial cell membranes, may be a target of interactions with peptides. The carbon chains of phosphatidylinositol molecules in its monolayer on an aqueous subphase are more rigid due to single carbon–carbon bonds, as compared to unsaturated compounds. At the molecular level, unsaturated double bonds hinder molecular packing, resulting in kinks in the hydrocarbon chain [41]. The addition of AA, EAA or P5 to the subphase influenced the properties of the PI monolayer. Due to the compression of the monolayer, the distance between the PI molecules decreased, and simultaneously, the strength of their mutual interactions increased. The recorded π-A isotherms differed slightly in the slope coefficient (a_LE/LC_) values in the range of the dynamic increase in the π-A isotherm surface pressure. The smaller the a_LE/LC_ value, the more feasible was the rearrangement of the PI molecules at the water/air interface, where they formed a packed monolayer [42]. The χ parameter can be considered an indicator of molecular compressibility, orientation changes and interactions between molecules during the compression of the monolayer. In the case of the PI monolayer on the aqueous subphase, it increased with increasing temperature. The lowest χ values were obtained in systems with P5 in the aqueous subphase. This would indicate the presence of P5 molecules between the PI monolayer molecules, which had a low tendency to form a monolayer at the water/vacuum interface because they contained nonpolar side chains that contributed to this effect. In this system, there was also a steric effect, which can be observed as a bulge at the surface of the film [43]. The interaction between the cationic peptide and the negatively charged part of phosphatidylinositol may add to this effect. For comparison, the χ parameter for azolectin and lecithin monolayers with P5 in the aqueous subphase was 0.078 and 0.072, respectively, at 25 °C, which was more than 0.2 times lower than the values obtained for the PI + P5 system under the same conditions [43]. The a_LE/LC_ value is also lower for the azolectin monolayer and lecithin monolayer compared to the PI monolayer with P5 in the aqueous subphase. This indicates an easier rearrangement of molecules at the water/air interface and thus the formation of a more tightly packed monolayer in the case of azolectin or lecithin. This is confirmed by the C_S_^−1^ values, which for the azolectin, lecithin and phosphatidylinositol monolayer were 64.7, 53.8 and 43.85, respectively [43]. Figure 7 presents the values of the following parameters of the tested systems at a temperature of 20–35 °C: A_collapse_ (Å^2^/molec.), π_collapse_ (mN/m) and C_S_**^−^**^1^.

It can be seen that the addition of P5 to the aqueous subphase in each system reduced the A_collapse_ (Å^2^/molec.) values. The collapse mechanism depends on several factors, including the structure of the polar head, the number of alkyl chains and the presence of other substances in the subphase. The PI monolayer contains several OH groups in its structure and can form hydrogen bonds with water molecules at the interface. P5, despite its low surface activity, contains hydrophobic chains in its structure, which can penetrate the alkyl chains of the phospholipid to some extent, according to similar research [44]. The collapse is dependent on the interaction of the monolayer with the subphase [45]. The surface pressure at which the multilayer collapses π_collapse_ (mN/m) decreases with increasing temperature; this is also the case after adding EAA and P5 to the water subphase. This is related to its elasticity, which increases with rising temperature. This may be the result of the greater stability of PI in the presence of these two compounds, which have a larger hydrophobic part in their structure than AA [46,47]. It should be remembered that the solubility of vitamin C changes with temperature, which has an additional impact on the physicochemical properties of the PI monolayer [48].

The R_v_ (%) coefficient value indicates the lowest reversibility of the compression process in the case of the PI + P5 system, which may be due to the strong interactions of PI molecules with P5. In other cases, the values vary depending on the tested system and temperature. The reversibility coefficient of isothermal compression in all systems increased in the successive hysteresis loops at most temperatures, which confirms the thesis that the interactions between the monolayer molecules are stabilized with the successive compression and decompression isotherm; this was also observed by other researchers who studied systems with chitosan and arachidic acid [49]. The flattening visible in Figure 5 for the PI monolayer and the PI + P5 + AA and PI + P5 + EAA systems in the aqueous subphase at temperatures of 30 °C and 35 °C could be a consequence of the phase transformation, revealed also in monolayers of silsesquioxane [50].

**Figure 8:**
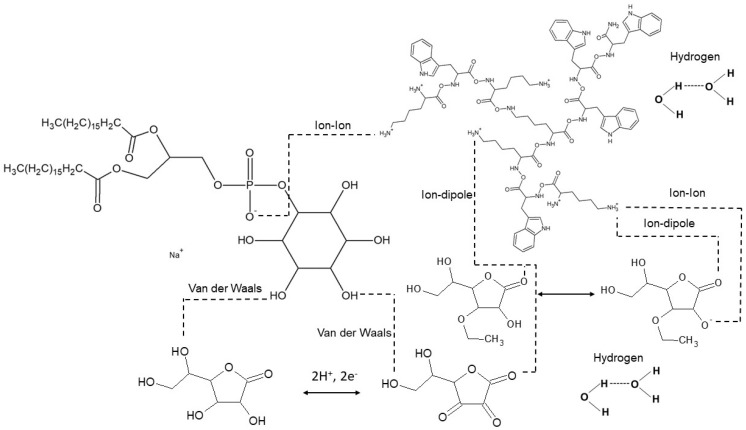
Examples of interactions between the tested compounds.

Based on the obtained results, we can assume that there are interactions between the compounds tested, as shown in Figure 8. Also, data obtained from FTIR and direct surface tension measurements collected by researchers who used another type of peptide support the concept of specific interactions between the peptides and EAA. Structural antimicrobial activity, toxicity and pharmacokinetic studies conducted to date have been aimed at assessing the effectiveness, safety and therapeutic potential of cationic peptides, both in the treatment of infections and in other medical applications.

## 3. Materials and Methods

### 3.1. Synthesis and Characterization of the Peptides

#### Preparation of the Peptides and Purity and Structure of the Peptides

The preparation, purification and determination of the structure of the peptides was carried out as described in our earlier publication [21]. Peptide compounds were manually synthesized by the Fmoc solid-phase (Iris Biotech, Marktredwitz, Germany) peptide synthesis method using Rink Amide AM resin (Iris Biotech, Marktredwitz, Germany, particle size 149–74 μm; loading 0.48 mmol/g). The amino acid coupling reaction was performed using DIC (Merck, Darmstadt, Germany) and HOBt (Merck, Darmstadt, Germany) activators with a threefold molar excess of each amino acid and activator dissolved in DMF/DCM (1:1; *v*/*v*, Merck, Darmstadt, Germany). Deprotection was performed using 20% (*v*/*v*) piperidine in DMF. The deanchoring of the peptides from the resin was achieved using a mixture of TFA/TIS/H_2_O (Merck, Darmstadt, Germany) in a volume ratio (95:2.5:2.5). The purity of the peptides was analyzed by reversed-phase high-performance liquid chromatography (RP-HPLC) on a Shimadzu Nexera chromatograph with a DAD detector at 214 nm equipped with Eurospher columns (100 × 4.6 mm) (Knauer, Berlin, Germany) using ACN:TFA (0.1%, Merck, Darmstadt, Germany and H_2_O:TFA (0.1%) as the mobile phase. The identity of the peptides was verified by matrix-assisted laser desorption time of flight (MALDI-TOF, Sciex, IL, USA) spectrometry on a MALDI-TOF/TOF 5800 instrument (Sciex, IL, USA). Peptides with identities confirmed by MS spectra were lyophilized (Christ, Hannover, Germany) and stored as dry powder at −20 °C.

### 3.2. Langmuir Films

To study monolayers formed of phosphatidylinositol (PI) on an aqueous subphase with P5 in the presence of AA (Fagron, Modlniczka, Poland) or EAA (Merck, Darmstadt, Germany), a Langmuir–Wilhelmy trough manufactured by Kibron Microtrough X in Helsinki (Finland), together with the accompanying FilmwareX 4.0 computer software, was used. The balance consists of a polytetrafluoroethylene Teflontray measuring 23.7 cm long and 7.9 cm wide, two movable Teflon barriers and a wire (used instead of a Wilhelmy plate) weighing 48.2 mg and 0.5 mm in diameter made of platinum, which ensures a negligible contact angle during the measurement. In order to avoid the introduction of impurities, before each measurement, the platinum plate was rinsed with methanol, then with water and ignited with a burner flame. The barriers moved at a speed of 10 mm/min. The cleanliness of the surface of the carrier phase was checked by measuring the surface tension during the movement of the railings towards the center of the tank. If the value of the voltage change did not exceed 0.30 mN/m, the surface was considered free of impurities. Otherwise, the washing procedure was repeated. The tubs were placed on anti-vibration tables. All measurements were performed with three repetitions. A Krüss thermostat (Krüss, Hamburg, Germany) was used to maintain a constant temperature of 20 °C, 25 °C, 30 °C and 35 °C during the measurements. The obtained results were processed using Microsoft Excel 2016.

### 3.3. Compression Isotherms of Phosphatidylinositol on the Aqueous Subphase with P5 and AA or EAA

After checking the purity of the subphase, chloroform (Merck, Darmstadt, Germany) and methanol (Merck, Darmstadt, Germany) were mixed in a ratio of 9:1,. and then phosphatidylinositol (C_47_H_82_NaO_13_P, Sigma-Aldrich, St. Louis, MO, USA) was dissolved in this mixture to obtain a concentration of 1.18 × 10^−6^ mol/L. The phosphatidylinositol solution prepared in this way was applied to the surface of the aqueous subphase. After the evaporation of the solvent, the monolayer was symmetrically compressed to a surface pressure of 5 mN/m. Then, 30 μL of an aqueous solution of the tested peptide at a concentration of 1.27 × 10^−3^ mol/L and 30 μL of an AA or EAA solution at a concentration of 1.27 × 10^−3^ mol/L were introduced into the subphase. After 15 min of system stabilization, the barriers were moved inwards at a speed of 10.02 mm/min. The force acting on the wire was calculated according to the formula given in previous paper [51].

### 3.4. Hysteresis

Isotherm compression–decompression was used analogously to measure isotherm compression. The same speed of moving and spreading the barrier was used (10 mm/min). However, in this case, the recorded measurement was for 3 loops. The isotherm compression reversibility factor was calculated according to Georgiev et al. [52].

### 3.5. Compressibility Coefficient of the Monolayer

Compressibility, denoted as C_S_, is a parameter that can be calculated from the course of the π-A isotherms commonly used to characterize monolayers. However, the concept of a compressibility modulus, compressibility factor or compressibility coefficient is more commonly used and defined as the reciprocal of compressibility [38].

### 3.6. Surface Pressure Changes over Time

A 15 μL phosphatidylinositol solution was applied to the aqueous subphase and left for 15 min for the solvent to evaporate. Then, the monolayer was compressed to the pressure range characteristic of natural biological membranes, i.e., 30 mN/m. Subsequently 30 μL of the antibacterial peptide under evaluation and 30 μL of AA or EAA were added to the aqueous subphase. Surface pressure was measured at selected temperatures, 25 °C and 35 °C, for the entire period of 60 min after the addition of the AA or EAA subphase as a function of time and constant surface area.

## 4. Conclusions

The molecular organization of the PI monolayer may change depending on the presence of ascorbic acid, 3-O-ethyl-L-ascorbic acid or P5 in the aqueous subphase and the system temperature. Due to the cationic nature of P5, it can interact with the anionic group of the phosphatidylinositol monolayer. The analysis of the compressibility coefficient shows that the most ordered monolayer was the PI + P5 + EAA system. With an increase in temperature, its value increased. The results of this study can be used in the process of designing new antimicrobial agents, especially those directed against *Cutibacterium acne*. It is also worth extending this study to microbiological tests on and an assessment of the anti-inflammatory properties of these systems.

## Figures and Tables

**Table 1:** The composition of the evaluated systems.

Evaluated Systems	Monolayer	Subphase		
	PI	AA	EAA	P5
PI *	1.20 × 10^16^	−	−	−
PI + AA *		2.30 × 10^16^	−	−
PI + EAA *		−	2.30 × 10^16^	
PI + P5		−	−	2.30 × 10^16^
PI + P5 + AA		2.30 × 10^16^	−	2.30 × 10^16^
PI + P5 + EAA		−	2.30 × 10^16^	2.30 × 10^16^

* Reference [37] reports a former evaluation of monolayers 1–3, which were reassessed for comparison purposes.

**Table 2:** Characteristic parameters of π–A isotherms: A_lift-off_—lift-off area of surface pressure; A_collapse_—area corresponding to monolayer collapse; π_collapse_—collapse pressure [mN/m]; χ (A_collapse_/A_lift-off_); a_LE/LC_—slope coefficient in range of dynamic increase in surface pressure of π–A isotherm.

Evaluated System	Temperature (°C)	A_lift-off_ (Å^2^/molec.)	A_collapse_ (Å^2^/molec.)	π_collapse_ (mN/m)	χ	a_LE/LC_
**PI**	20	101.35	13.50	38.43	0.13	−0.571
	25	101.27	17.09	38.89	0.17	−0.594
	30	101.30	21.56	35.06	0.21	−0.573
	35	101.26	21.38	35.08	0.21	−0.572
**PI + AA**	20	101.53	24.11	37.96	0.24	−0.588
	25	102.80	33.73	21.23	0.33	−0.552
	30	101.54	18.23	27.67	0.18	−0.529
	35	101.54	34.00	32.41	0.33	−0.547
**PI + EAA**	20	100.72	13.50	41.74	0.13	−0.581
	25	101.10	22.05	38.46	0.22	−0.586
	30	101.86	33.27	35.11	0.33	−0.558
	35	101.60	13.68	29.97	0.13	−0.547
**PI + P5**	20	100.72	10.55	44.41	0.10	−0.588
	25	98.34	9.41	36.46	0.10	−0.549
	30	98.13	5.00	39.01	0.05	−0.559
	35	97.92	9.06	37.15	0.09	−0.553
**PI + P5 + AA**	20	101.70	33.58	25.85	0.33	−0.545
	25	100.53	12.70	26.19	0.13	−0.403
	30	102.36	10.79	25.01	0.11	−0.569
	35	101.83	11.21	29.21	0.11	−0.540
**PI + P5 + EAA**	20	101.76	11.21	39.45	0.11	−0.559
	25	102.06	12.51	34.45	0.12	−0.546
	30	102.12	14.31	29.09	0.14	−0.578
	35	102.40	10.02	24.29	0.10	−0.575

**Table 3:** Compressibility coefficient C_S_^−1^ [mN/m] values of the tested systems.

Evaluated Systems	20 °C	25 °C	30 °C	35 °C
PI *	46.23	48.18	48.22	49.95
PI + AA *	49.39	51.58	47.53	44.60
PI + EAA *	45.47	49.01	46.77	47.21
PI + P5	45.39	43.85	45.87	45.47
PI + P5 + AA	48.68	35.28	54.32	51.50
PI + P5 + EAA	45.97	49.53	52.28	57.49

* Reference [37] reports a former evaluation of monolayers 1–3, which were reassessed for comparison purposes.

**Table 4:** Isotherm compression reversibility coefficient R_v_ (%) for loops 1–3 of PI monolayers with P5, AA and EAA peptides in the aqueous subphase.

Temperature			R_v_ (%) Parameter for the Evaluated Systems				
		PI	PI + AA	PI + EAA	PI + P5	PI + P5 + AA	PI + P5 + EAA
20 °C	loop 1	70.77	70.13	72.84	80.59	68.75	77.24
	loop 2	73.06	81.89	80.04	88.67	78.96	86.20
	loop 3	72.66	83.64	82.32	96.43	80.76	85.04
25 °C	loop 1	74.54	73.30	72.63	87.34	71.75	73.51
	loop 2	77.57	77.64	80.30	90.32	78.46	79.72
	loop 3	79.03	79.60	81.95	87.11	77.11	81.36
30 °C	loop 1	86.81	59.59	67.03	83.47	72.62	73.35
	loop 2	77.23	73.46	76.80	97.06	71.13	77.70
	loop 3	80.80	79.49	80.61	95.94	73.20	86.87
35 °C	loop 1	66.43	51.74	60.53	87.34	69.49	77.60
	loop 2	79.77	69.83	74.97	93.87	68.66	75.34
	loop 3	86.83	74.38	78.56	97.77	68.60	70.59

## Data Availability

The scientific data are available from Wroclaw Medical University, Department of Physical Chemistry and Biophysics.
